# Effects of Habitual Anger on Employees’ Behavior during Organizational Change

**DOI:** 10.3390/ijerph10126215

**Published:** 2013-11-25

**Authors:** Mareike Bönigk, Georges Steffgen

**Affiliations:** Integrative Research Unit on Social and Individual Development (INSIDE), University of Luxembourg, Campus Walferdange, Route de Diekirch, Walferdange L-7201, Luxembourg; E-Mail: boenigk@pt.lu

**Keywords:** organizational change, anger reactions, employee well-being, resistance to change

## Abstract

Organizational change is a particularly emotional event for those being confronted with it. Anger is a frequently experienced emotion under these conditions. This study analyses the influence of employees’ habitual anger reactions on their reported behavior during organizational change. It was explored whether anger reactions conducive to recovering or increasing individual well-being will enhance the likelihood of functional change behavior. Dysfunctional regulation strategies in terms of individual well-being are expected to decrease the likelihood of functional change behavior—mediated by the commitment to change. Four hundred and twelve employees of different organizations in Luxembourg undergoing organizational change participated in the study. Findings indicate that the anger regulation strategy venting, and humor increase the likelihood of deviant resistance to change. Downplaying the incident’s negative impact and feedback increase the likelihood of active support for change. The mediating effect of commitment to change has been found for humor and submission. The empirical findings suggest that a differentiated conceptualization of resistance to change is required. Specific implications for practical change management and for future research are discussed.

## 1. Introduction

Following the general shift of attention by psychological researchers from cognitive to affective processes [1], an increase in the number of studies focusing on the role of emotions during organizational change, the interaction of emotions with cognitions, and the impact of emotions on change-behavior has been observed [2]. This increase in research on emotion in organizations led to the integration of complex psychological processes in the modeling of organizational change processes [3]. Emotions are no longer seen as interrupting well-structured processes but as providing necessary information to effectively deal with complex social situations [4]. 

Many studies on innovation and organizational change still rather emphasize organizational-level factors based on a top-down perspective of implementation. The individual-level, bottom-up processes of innovation have often been ignored, even though many change-implementations fail due to a lack of acceptance and realization by those confronted with the change [5]. As a consequence, researchers in the field state that further research is required focusing on the role of emotions for differential behavior during change [6]. 

### 1.1. Background

The well-established Affective Events Theory (AET) [7] theoretical framework related to affect in the workplace serves as a model for our study. AET supports the idea that singular work events influence long term workplace behavior through emotional states or, more specifically, emotion regulation strategies. Workplace behaviors are conceptualized to include change specific performance.

Organizational change is generally considered to have the strongest potential to trigger emotions compared to other work events [8]. Organizational change can be defined as a stressful process, which can be perceived as a threat or a challenge by the individual. In this context, the assessment of the change event will be influenced by the impossibility to anticipate outcomes of processes under change, by interpersonal or inter-role conflicts because of unclear roles within new organizational processes, by perceived injustice or perceived loss of control [6]. These evaluations will impact the emotions that occur and according to AET, these emotions in turn will influence behavior during change [9].

The first question in this context is which emotion is especially relevant during change. Explorative studies show that fear, but also contentment or pride are often reported. These are therefore considered relevant emotions during organizational change. Other studies point out that anger can be assumed to be especially relevant in our context of interest [10]. Individuals report anger most likely when their goals have been thwarted, individual or group norms have been violated or injustice is perceived [11]. Anger is likely to play an important role in the change process for several reasons: firstly, there is the change inherent risk to frustrate individual goals (by changing tasks and processes), secondly, there is the risk of lack of support (for instance because of managerial overload during change), thirdly the risk of higher work load, and finally endangered social relationships (by restructuring work groups or layoffs). The statement that anger is a relevant emotion during organizational change alone does neither hold an inherent assumption about the quality of behavior during organizational change, nor about its functionality. These factors need to be analyzed and specified in the light of the individual’s anger reaction and situative variables, especially focusing on a multidimensional concept of resistance to change [12]. 

### 1.2. Objectives

Anger is a multidimensional emotion that involves an appraisal of responsibility for wrongdoing by another person or entity and often includes the goal of correcting the perceived wrongness [13]. Anger reactions bear functional and dysfunctional implications for work life and performance as well as for individual well-being. Traditionally, research on anger, and even more so in the organizational context, focuses on the maladaptive consequences of anger and thus the negative aspect of anger [14]. Lately, we witness an increased interest in a social functional approach to anger—an approach that is still underrepresented in organizational research [15,16]. 

As we are interested in the impact of change related anger on change behavior, we focus on the multidimensionality of anger reactions: Is anger rather related to aggression, work related stress, raised blood pressure, less cooperation and productivity or more to increased determination, preparedness to take responsibility and problem solving competencies? The answer can only be elucidated in the light of anger regulation strategies and under consideration of the characteristics of the situation in question [17]. 

With the objective to establish or enhance individual well-being, the functionality of anger regulation strategies refers to the extent to which they are likely to: (a) change the sources of anger and reduce the likelihood of future incidents, (b) reduce feelings of anger, and (c) to dampen physiological reactivity [18]. Functional regulation strategies include providing feedback, downplaying the incident’s negative impact, humor or distraction. Dysfunctional anger regulation strategies leading to negative outcomes for the individual’s well-being comprise venting, submission or rumination [19]. It is important to note that an anger reaction, conducive for recovering or increasing individual well-being and that is therefore perceived as being beneficial by the individual, does not have to be functional in terms of performance or organizational outcomes at the organizational level as we will exemplify further down. 

Under a multidimensional conceptualization of anger reactions an individual’s anger reaction does not automatically lead to deviant change resistance but opens a broad range of possible reactions as a function of the individual anger reaction. Conversely, Active change support does not preclude that the protagonist did not feel angry during the change process or because of it. The objective of this study is to add to the understanding of these seemingly contradictory interrelationships. 

Three research questions concerning behavior during organizational change guided our research:

(1) Which habitual anger reactions potentially contribute to functional change behavior in the organizational perspective and which habitual anger reactions are likely to act as obstacles for organizational change?
(2) Where do we find contradictions regarding individual and organizational interests?
(3) How can we add to a differentiated conceptualization of resistance to change?

**Figure 1:**
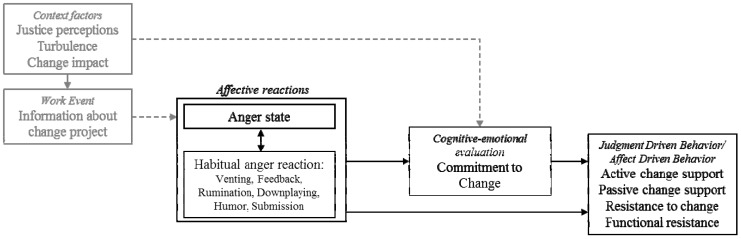
Integration of the hypotheses based on the redrawn AET-model (see [20], p.23).

Furthermore, the influence of other facilitators or barriers explaining stated behavioral differences will be integrated in our analysis (such as context factors or change related attitudes) to allow for some of the complexity in our research question (see Figure 1 for an integration of the aforementioned interdependencies).

## 2. Methods

### 2.1. Data Source and Study Population

This study was conducted in four Luxembourg-based organizations undergoing organizational change and supplemented by an open survey (EFS Survey 6.0, Globalpark AG, Köln, Germany) spread per e-mail in business-networks in Luxembourg leading to an overall sample of N = 412 employees in Luxembourg. As a direct consequence of the financial crisis, which also affected Luxembourg-based companies, acquisition of participating organizations was particularly difficult and took 17 months. The survey itself was limited to two weeks within each organization. Respondents were asked to provide socioeconomic characteristics, including age, sex, hierarchical status, seniority, workplace, and nationality. Age varies from under 30 years to over 60 years with 27% aged between 30 and 40 years. Seniority ranges from 2 to more than 15 years (22% for the latter). 18% state that they are working in a management position. Following the typology for organizational changes of Porras and Robertson [21], 35% of the participants are undergoing structural change within their organization, while 27% are facing the implementation of short time work. 23% are ask to implement new technology. Other changes are the removal of the company or a change within social factors.

### 2.2. Hypotheses

#### 2.2.1. Habitual Anger Reactions

*Venting*: In contrast to the catharsis hypothesis [22], venting is perceived as dysfunctional for anger coping and therefore also for individual well-being. Under this perspective venting refreshes the anger event and escalates conflict rather than reinforcing conflict resolution [23]. In the context of organizational change, venting is likely to violate organizational norms for anger expression [16,24] and is appraised as dysfunctional change behavior. In this perspective, venting is expected to have a positive impact on deviant resistance to change.

*Feedback*: Feedback is considered an expressive form of anger reaction and characterized as “constructive” ([25], p. 55), as a manner to directly influence and change anger evoking conditions that also positively influence physiological arousal [25]. Feedback, like venting, can be understood as “social species of reappraisal” ([26], p. 143). This social approach discloses social resources that help the individual to find efficient ways for anger regulation when solution oriented. In the light of humanistic management approaches, feedback can be seen as a basis for participation and can be understood as functional, even as active support of change. However, if the organization does not apply a participative management approach, feedback can be understood as active resistance. In both cases, the influence of feedback should be mediated by commitment to change as the cognitive—emotional evaluation will be the basis for problem solution manifested by feedback.

*Humor*: Humor, or more precisely coping-humor, is—as well as *downplaying*—understood as a form of cognitive reappraisal. As already established in the psychoanalytic tradition humor thus enhances individual well-being, increases social attraction of the humorous individual and positive affect in general and reduces physiological arousal [27]. More recently, humor has been defined as a multidimensional construct, consisting of affiliative, self-enhancing, aggressive and self-defeating dimensions [28]. These latter two dimensions have more negative implications—especially within working context [29]. Taking into consideration the positive consequences of coping-humor as a strategy for cognitive reappraisal, we hypothesize a positive influence of humor on change support, mediated by commitment to change. 

*Downplaying*: Reappraising the potentially anger evoking situation is expected to also have a positive influence on supportive change behavior as it enhances individual well-being and therefore openness for dealing with the change. 

*Rumination*: Lybumirsky and colleagues ([30], p. 1059) conclude that dysphoric rumination drains energy and motivation, interferes with efforts to take the needed steps in problem solving, may become self-perpetuating and thus intensifies negative mood. Chronic rumination increases the risk of cardiovascular diseases [31] an effect that is stronger for women who tend to ruminate more than men [32]. Rumination can be an expression of learned helplessness. The emergence, upkeep and consequences of burnout, chronic pain and stress have been discussed in this light [33]. Taking these findings into account, we predict active change support to be less prevalent in those individuals who tend to habitually ruminate while passive support or passive resistance might be positively influenced by habitual rumination.

*Submission*: Submission is defined here as special case of suppression [34] which is especially important at the work place. The authors see submission as motivation to suppress anger—leading in the end to the same consequences. These are mostly negative for the individual well-being: The “silent anger” [35] is exhausting, weakens body´s defenses, leads to rumination and raises blood pressure [36]. Although rather dysfunctional for the individual well-being, the organization might define this anger reaction as functional change behavior as it does not lead to resistance against change. We suggest that submission as habitual anger reactions makes passive support of change more likely. 

#### 2.2.2. Change Related Cognitive and Emotional Evaluation—Commitment to Change

As suggested by Russell and Eisenberg [20] the AET-model includes the cognitive-emotional evaluation of the as complement of the event related attitude. We therefore integrated commitment to change as result of the cognitive-emotional evaluation of the organizational change event here. Following Herscovitch and Meyer’s [37] arguments, commitment to change is one of the most important factors involved in employees’ support for change initiatives. Their commitment-concept is composed of three commitment-dimensions—affective, calculative and normative commitment. In this paper we will focus on the first two commitment dimensions. Affective commitment to change reflects “a desire to provide support for the change based on a belief in its inherent benefits” [37] and should therefore have a positive impact on supportive change behavior. In parallel, affective commitment might also prevent deviant resistance to change. Calculative commitment to change refers to employee support for change without being persuaded. The employee supports change because he or she wants to prevent anticipated negative consequences of resistant behavior. Calculative commitment to change is expected to have the same implication as affective commitment to change, but the effects should be weaker. According the implications of AET, commitment might mediate the impact of habitual anger reactions on change behavior.

(1) The hypotheses concerning habitual anger reactions can be summed up as follows, resistance to change is expected to be higher in employees who tend to vent their anger (1a) and—depending on feedback culture of the organization—who tend to give feedback as habitual anger reaction (1b). The influence of feedback is likely to be mediated by commitment to change (1c).
(2) Active change support is more likely when employees habitually tend to give feedback when angry (2a)—if feedback is welcome in the organization in question. Habitually reappraising anger by reacting humorously to anger evoking situations (2b) or by downplaying the incident (2c) are also predicted to have a positive influence on active change support. All influence on active change support are be mediated by commitment to change (2d).
(3) Passive change support might also be enhanced by cognitive reappraisal (humor, (3a) and downplaying, (3b)) as well as by rumination (3c) and submission (3d) as outlined above. Again, the influence will be mediated by commitment to change (3e).

#### 2.2.3. Context Factors

To capture context- and process-variables, which, according to AET determine the affective evaluation of an event, we integrated the perceived impact of change, justice perceptions and the perceived turbulence of the change environment in our study. This additional step respects the complexity of organizational change influencing individual change behavior. 

In this context, turbulence refers to “(…) the preponderance of changes going on in the organization at the same time as the focal change—changes that represent additional distractions and adaptation demands and thus form an important part of the context for individuals’ reactions to the focal change” [38]. 

Research in organizational justice perception has shown that when organizational decisions and managerial actions are deemed unfair, the affected employees experience feelings of anger, outrage and a desire for retribution [39]. Both perceptions of procedural [40] and interactional [41] injustice have been associated with feelings of anger among employees. Distributive justice perception being an important anger eliciting appraisal is more connected to change outcomes than to process characteristics and is therefore not considered here. 

The perception of major organizational change and also the perception of a turbulent change environment enhance uncertainty and ambiguity [42] which in turn impair a positive affective commitment to the change and somewhat strengthen risk awareness. The latter should lead to calculative commitment to change. Based on our theoretical assumptions we also expect a positive influence of perceived change impact as well as turbulence on anger state. 

Interactional and procedural justice perceptions have a negative impact on anger state and a positive impact on affective commitment to change but should not add to the understanding of calculative commitment to change which is more related to the fact of change *per se* and less to the content or the way the change is carried out [37]. 

### 2.3. Outcome Measures

Based on the results of a preliminary study it was determined that an important affective event in the context of organizational change is the first information about the future change given by those implementing the change [43]. This first change announcement served as affective event in the present study.

#### 2.3.1. Anger State, -Reactions and -Goals

Anger state is measured retrospectively by STAXI-2 [44] (6 items, α = 0.91; Range: 6–24; *M* = 9.78; *SD* = 5.15). The individual, habitual anger reaction at the workplace (28 items) and the related habitual goals (28 items, 4-pt-Likert-scale) have been measured by the “Anger Reactions and Goals Scale” (ARGI) [19,45] which has been adapted to the work-context. Dimensions for anger reactions within the ARGI are venting, feedback, ruminating, humor, downplaying, submission, and distraction which fits in with the multidimensional concept of anger applied here (α = 0.69–0.83). Goals of habitual anger reactions are either avoidance intended or approach intended (α = 0.68–0.81).

#### 2.3.2. Commitment to Change

Individual normative, affective and calculative commitment to change is measured by a slightly shortened version (12 items) of the Commitment to Change-Scale by Herscovitch and Meyer [37] (4-pt-Likert-scale; α = 0.62–0.83).

#### 2.3.3. Self-Reported Behavior during Change

The self-reported outcome measure “behavior during change” is based on the work of Herscovitch and Meyer [37]. The authors understand behavior during change as a continuum defined at one end by “championing” which stands for extra-role performance, followed by “cooperation” and then by the more passive “(mere) compliance” as sort of minimalistic cooperation during organizational change. On the other end of this continuum, Herscovitch and Meyer [37] see passive resistance as: “(…) demonstrating opposition in response to a change by engaging in covert or subtle behaviors aimed at preventing the success of the change”. And finally active resistance as: “(…) demonstrating opposition in response to a change by engaging in overt behaviors that are intended to ensure that the change fails”.

To measure behavior during change we used Herscovitch and Meyer’s [37] items supplemented by our own items resulting in a scale that consists of 21 items (5-Likert-scale; α = 0.31 (compliance)–0.82).

Because of unsatisfying internal consistencies and also because of theoretical implications (if behavior during change is viewed as a continuum, active change support and active resistance should load on the same factor with opposite signs which contradicts our conception of resistance to change), we tested the factor structure of this scale. The factor analysis results in four factors (α = 0.43–0.81: Active support—reflecting discretionary behavior as suggested by Herscovitch and Meyer [37] and passive support—can be seen close to the compliance-dimension. The third factor, consisting of three mere compliance-items and three resistance-items is interpreted here as resistance factor and finally the fourth factor, consisting of two active resistance-items and two championing-items is interpreted here as functional resistance.

#### 2.3.4. Potential Context-Factors of the Affective Event

To survey the perceived impact of the change, the 6-item-scale “Individual Job Impact” [46] (α = 0.86; range: 6–30; *M* = 21.22; *SD* = 6.24; 5-pt-Likert-scale) has been applied. The process variable “procedural and interactional justice perception” has been measured by a scale developed specifically for justice perceptions during organizational change by Paterson, Green and Cary [47] which has been supplemented by two items to measure Leventhal’s [48] concept of process correctiveness (procedural justice perception: 10 items; α= 0.93; range: 9–45; *M* = 26.52; *SD* = 9.60; interactional justice perception: 6 items; α = 0.93; *M* = 18.34; *SD* = 6.89). The perceived environmental turbulence was imposed with the help of Herold, Fedor and Caldwell’s [38] 3-item-scale (α = 0.70; range: 3–15; *M* = 10.30; *SD* = 3.39).

### 2.4. Statistical Analysis

After analyzing group effects, the above anger reaction and change behavior measures were summarized for the whole sample. In addition to binary statistics, we also conducted multivariate analyses for selected outcome measures to examine if any difference existed in change behavior, based on: (1) habitual anger reactions, and (2) commitment to change as well as to (3) investigate the influence of context factors on commitment to change and anger state. All group comparison and regression models were estimated by IBM^® ^SPSS^® ^Statistics 19.0.0. 

## 3. Results and Discussion

We first report group differences, followed by a presentation of the descriptive and multivariate analysis of the habitual anger reactions and change behavior, respectively. Additionally, the influence of the context factors on anger state and on commitment to change will be presented.

### 3.1. Group Comparisons

Group differences were evaluated using an Independent T-test or ANOVA. All group effects found correspond to the outlined theoretical assumptions and therefore strengthen the quality of data and measures.

First of all, we assume small but significant differences between the different participating organizations and the participants of the open survey regarding all context factors and also commitment to change which totally correspond to our hypotheses. Those who perceive higher impact and turbulence show significantly higher calculative commitment to change. 

Additionally, we find small effects from seniority on calculative commitment to change in a way that those working more than 15 years for the organization in question show higher calculative commitment to change than with less seniority (*M_min_* = 11.02, *SD* = 4.82; *M_max_* = 14.24, *SD* = 4.34; *F*_(4,293)_ = 4.81, *p* < 0.01, *r_yλ_* = 0.331). These interdependences also correspond to our considerations in Section 2.2.2 because of higher risk awareness caused by weaker employability at rising age [49] and because of increasing transactional contracts leading to calculative commitment [50]. 

Those occupying a leadership position (*n* = 74 including team leaders) perceive higher justice perception compared to employees, especially procedural justice perception (*M_L_* = 28.82; *SD* = 9.27; *M_E_* = 24.9; *SD* = 9.39; *F*_(2,268)_ = 4.35, *p* < 0.05, *r_yλ_* = 0.206) and report a significantly higher affective commitment to change (*M_L _*= 16.26; *SD* = 3.87; *M_E_* = 14,33; *SD* = 4,09; *F*_(2,268)_ = 5.86, *p* < 0.01, *r_yλ _*= 0.236; see Bruning and Snyder [51] for corresponding results). Still focusing on hierarchical differences, we can show that workers state a stronger calculative commitment to change compared to leaders and employees (*M_W_* = 15.4, *SD* = 3.65; *M_E_* = 13.11, *SD* = 3.75; *M_L_* = 12.03; *SD* = 4.46; *F*_(2,267)_ = 13.5, *p* < 0.001, *r_yλL-W _*= 0.388, *r_yλE-W _*= 0.303) and that they more likely show humor in reaction to anger than leaders (*M_W_* = 5.46, *SD* = 1.85; *M_L_* = 4.27, *SD* = 1.79; *F*_(2,272)_ = 6.99, *p* < 0.001, *r_yλ _*= 0.311). In consequence, leaders report different change behavior compared to employees and workers—less resistant behavior, more functional resistance to change, more active support which reflects their different role within change (see ([52], p. 136) for comparable results).

Other group differences for habitual anger reactions are based on gender. Men (*M* = 4.54, *SD* = 1.84) have a slightly higher tendency to vent their anger than women (*M* = 4.16, *SD* = 1.38; *t* = 2.05, *p* < 0.05, *df* = 363, *r_yλ_* = 0.107) and tend to ruminate less over the anger incident (*M* = 6.66, *SD* = 2.39) than women (*M* = 7.25, *SD* = 2.35; *t* = −2.23, *p* < 0.05, *df* = 363, *r_yλ_* = 0.116). 

As all group differences can be understood as effects of the hypothesized interdependences of our variables under study, we further on analyze the influences of habitual anger reactions on change behavior for the whole sample. 

### 3.2. Determinants of Self-Reported Change Behavior

#### 3.2.1. Resistance to Change

We hypothesized that the variance of resistance to change will be partly explained the habitual anger reaction venting (1a) and feedback (1b) and by commitment to change (1c). As feedback does not correlate with the criterion and humor does (*r* = 0.233, *n* = 375, *p* < 0.001), the regression model has been post hoc corrected. 

The resulting model explains 26.9% of total variance of resistance to change, *F*_ (4,360)_ = 33.12, *p* < 0.001. Affective commitment to change has a negative influence as predicted (β = −0.36, *p* < 0.001) while calculative commitment has a positive effect on resistance to change (β = 0.18, *p* < 0.001)—which has not been expected. The habitual anger reaction venting positively predicts resistance to change (β = 0.16, *p* < 0.01). In addition, we find a significant correlation between rumination and venting (*r* = 0.24, *p* < 0.001). Unexpectedly, the habitual anger reaction humor also has a positive impact on resistance to change (β = 0.17, *p* < 0.001). 

Our research model suggests that the influence of habitual anger reactions on self-reported change behavior might be mediated by commitment to change. We tested this assumption by applying hierarchical regression analysis [53] in Table 1.

**Table 1:** Mediation analysis (affective commitment) ^a^.

	Step 1	Step 2	Step 3	Step 4
DV:	Model 1:	Model 2:	Model 3:	Model 4:
	resistance to change	affective commitment	resistance to change	resistance to change
**Predictor**				
Humor	0.23 ***	−0.15 **		0.17 ***
				
**Mediator**				
Affective Commitment			−0.43 ***	−0.40 ***
				
***F***	21.40 **	8.34 **	81.24 ***	48.79 ***
***R^2^***	0.05	0.02	0.18	0.21
***Adjusted R^2^***	0.05	0.02	0.18	0.21
***df***	1	1	1	2

^a^ Beta for the last step in each model. ** *p* < 0.01; *** *p* < 0.001.

Sobel-test [54] shows that the mediation-effect from affective commitment to change to humor and resistance to change is significant (*z* = 2.74, *p* < 0.01) so that the habitual anger reaction humor increases the likelihood for resistance to change if affective commitment is low. 

#### 3.2.2. Active Change Support

Active change support should be positively influenced by the habitual anger reactions feedback (hypothesis 2a), humor (2b) and downplaying (2c). High commitment to change should have a positive influence on supportive change behavior and should mediate the influence of habitual anger reactions (2d). 

Calculative commitment to change (*r* = −0.08) and humor as habitual anger reaction (*r* = −0.08) do not significantly correlate with active support and are therefore excluded from regression analysis. The assumed positive impact of the habitual anger regulation strategy humor on active change support is therefore not reflected by our data and will be discussed later on in this paper. We find a negative correlation of venting and active change support (*r* = −0.12, *p* < 0.05).

The *post hoc* corrected multiple regression analysis therefore includes affective commitment to change, feedback, downplaying and venting as potential predictors for active change support. Our resulting model explains 35.7% of variance, *F*_(4,361)_ = 50.21, *p* < 0.001. As expected (2a), we found that the habitual anger reaction feedback positively predicts active support (β = 0.18, *p* < 0.001) underlining the positive social function from feedback as developed by Geisler, Wiedig-Allison and Weber [55]. Habitually downplaying anger also contributes to the explanation of variance within active change support (β = 0.14, *p* < 0.01; hypothesis 2c). Affective commitment to change (β = 0.53, *p* < 0.001) influences active support positively supporting Herscovitch and Meyer’s [37] conceptualization of the construct, but does not mediate the influence of none of the habitual anger reactions in our model. Habitual venting does not significantly influence active change support (β = −0.03, *p* = 0.43). 

#### 3.2.3. Functional Resistance to Change

The new resistance dimension which we called functional resistance to change represents behavior during organizational change that does not support the change as suggested by management and can therefore be called resistance. But functional resistance change does not reflect pure opponent behavior, it rather aims to adapt or optimize the change project which implies a basic commitment to the change. Based on these assumptions, we hypothesized a positive influence of the habitual anger reaction feedback which should be part of a functional resistance to change. The habitual anger reaction submission should in exchange lower the likelihood of functional resistance to change. 

The total explained variance for functional resistance to change by feedback, submission and affective commitment to change is 12%, *F*_(3,363)_ = 16.45, *p* < 0.001. Only feedback has a positive influence on functional resistance to change within our model (β = 0.32, *p* < 0.001), while affective commitment to change and submission have no significant impact (β = 0.09, *p* > 0.05 for affective commitment; β = −0.04, *p* > 0.05 for submission).

#### 3.2.4. Passive Change Support

Based on the considerations outlined above, passive change support should be positively influenced if employees habitually tend to cognitively reappraise their anger (by humor, 3a, or by downplaying, 3b) or if they tend to ruminate (3c) or to submit to the anger evoking conditions (3d). Again, commitment to change should be positively related to passive change support (3e). Only affective commitment to change correlates with passive change support (*r* = 0.31, *p* < 0.001). 

#### 3.2.5. Context Factors

With the help of stepwise regression we tested how far the context variables interactional and procedural justice perception, turbulence and change impact influence the retrospective anger state at the moment of the first change announcement. Based on this analysis (see Table 2) we conclude that interactional justice perception as well as the turbulence of the change environment significantly predict the anger state (β = −0.19, *p* < 0.01 for interactional justice perception; β = 0.13, *p* < 0.05 for perceived turbulence).

**Table 2:** Stepwise regression from context factors to anger state ^a^.

DV:	Model 1: anger state	Model 2: anger state
**Predictors**		
Impact	0.21 ***	0.10 ^†^
Proc. justice perc.	−0.17 **	−0.07
Interact. justice perc.		−0.19 **
Turbulence		0.13 *
***F***	15.84 ***	12.14 ***
***R^2^***	0.08	0.12
***Adjusted R^2^***	0.08	0.11
***df***	2	4
***N***	353	353

^a^ Beta for last step in each model. ^†^ *p* < 0.10; * *p* < 0.05; ** *p* < 0.01; *** *p* < 0.001.

This weakening of procedural justices influence in favor of interactional justice perception strengthen Weber’s [11] understanding of anger as social emotion being preliminary provoked by interaction with others. So, interactional injustice perception provokes anger more than perceived procedural injustice. Another explanation for the comparably weaker impact of procedural justice lies in the broadness of the construct itself: Ambivalent judgments may relativize procedural justice perception in sum. Finally, Rodell and Colquitt [56] point out that substantial information regarding procedural justice is less accessible than information for interactional justice assessment leading to smaller impact on behavior during organizational change. 

In correspondence with current research [57], affective commitment is determined by procedural (β = 0.19, *p* < 0.01) and interactional justice perception (β = 0.35, *p* < 0.001) and by perceived turbulence (β = −0.11, *p* < 0.05) (*R^2 ^*= 0.26, *F*_(4,348) _= 29.73, *p* < 0.001). The higher justice perceptions and the lower turbulence is perceived in the change environment, the more likely is an affective commitment to change of those being confronted with it. 

Calculative commitment to change is significantly influenced by the context factor turbulence (β = 0.18, *p* < 0.01; *F*_(4,353)_ = 10.53, *p* < 0.001) so that that sort of commitment is more likely in situations perceived as insecure. As expected, justice perceptions do not significantly influence calculative commitment to change in the model.

Change impact does not contribute to the prediction of neither anger state nor affective commitment to change and only marginally contributes to the prediction of calculative commitment to change (β = 0.12, *p* < 0.10) which contradicts Caldwell, Herold and Fedors [46] findings as well as those of Rafferty and Griffin [58] and our expectations based on these studies. 

Thus, summing up our findings, especially interactional justice perception and also the perceived turbulence of the change environment determine the intensity of change related anger state and the individual commitment to change. 

### 3.3. Discussion

Our research interest reflects Poder’s [59] as well as Szasz, Szentagotai and Hofmann’s [60] request to focus on origin and informational value of anger during change—especially on interindividual differences in habitual anger regulation. Furthermore, we investigated context factors that can be understood as barriers or facilitators of active change support. 

#### 3.3.1. The Role of Habitual Venting

As hypothesized, those individuals who tend to vent their anger (based on our findings more likely men and workers), are more likely to (dysfunctionally) resist against the change. Within the context of most organizations, such a behavior will be interpreted as deviant from cultural norms [16,24] and as something one should eliminate. It is less likely that the organization will be able and willing to use the information value of this resistance to change. Venting and the implied violation of organizational norms is, as was argued above, likely to harm individual well-being. At individual level, training and therapy should focus on changing the habitual anger reaction to increase well-being while in parallel at organizational level, the information value of venting should be recognized. Possibilities should be explored to recognize and accept resistance as information and offering employees a means to functionally resist change [61]. 

#### 3.3.2. The Role of Habitual Feedback

Employees, who tend to verbalize their anger in a solution oriented manner [25], also risk to be understood as being resistant—depending on cultural norms within the organization, status or interaction partner. Such a resistance is nevertheless most probably functional as the solution orientation guarantees optimization rather than to hindrance of change. Even though our findings have to be seen as explorative, our approach underlines the necessity of a multidimensional understanding of resistance to change. 

Further research is necessary to understand the underlying processes, in particular the relationship between commitment to change and feedback. Contrary to our hypothesis, we could not find a significant correlation between commitment to change and feedback. One possible explanation for this is our operationalization of the habitual anger reaction as a general expression of the willingness to participate in decision making rather than an explicit situation related intend. This interpretation is underlined by the higher feedback rate within management in our study. For future studies we suggest to correlate habitual feedback to other personality traits such as extraversion or locus of control. For applied psychology we recommend to integrate the need to give feedback in assessment techniques when being precise about participation possibilities.

#### 3.3.3. The Role of Habitual Rumination

Even though we could not show a direct influence from habitual rumination on change behavior, we consider the positive correlation with both venting and submission as reinforcement for our reflections regarding the consequences of habitual rumination. Knowing about the potentially negative impact of rumination on individual well-being and considering the above mentioned correlations, management should consciously try to interact with the employees applying this strategy. Such a social support should aim to dissolve circular negative thinking, for instance by anticipating potential outcomes of the change and by dealing offensively with uncertainty. Involving ruminative employees may also impair ruminative thoughts. 

#### 3.3.4. The Role of Habitual Downplaying

While feedback as “social species of reappraisal” ([26], p. 143) attempts to (inter-) actively change the situation, habitual downplaying helps to cognitively change the appraisal of the situation. Both approaches are characterized by a problem-solving attitude. Analysing hypothesis 2c, we found evidence that downplaying has a positive impact on active change support. So it might be fruitful for change management to support employee´s individual striving for cognitive reappraisal in the direction of downplaying. This does not mean that management should minimize or hide negative, ambivalent or unclear factors of the change. Transparent, continuous communication is inevitable to hinder cynic reactions, strengthen trust and gain affective commitment to change [62]. Downplaying in a functional way focuses on respecting employee’s fears, on cooperative relativizing and on solution finding.

#### 3.3.5. The Role of the Habitual Anger Reaction Humor

As our results show consequently non-hypothesized influences for humor, this needs an in-depth discussion. Following the argumentation of Ray and colleagues [26], should reappraisal of the situation that is humor-inherent, help to overcome anger and foster performance. Contradictory, the habitual anger reaction humor increases here the likelihood of deviant resistance to change, mediated by affective commitment to change (the lower affective commitment to change, the higher humor). These findings question the interpretation of the ARGI-R humor scale by Kubiak and colleagues [19] and suggest that this scale should be rather interpreted as change related cynicism [63]. Cynicism is an often shown reaction in the context of organizational change as such changes are often characterized by value-conflicts [64], individual failure [65] or a perception of incompetence, laziness or a lack of integrity within others [66]. Additionally, unrealistic positive change propaganda enhances cynic reactions from employees [67]. Social cynicism is related to less cooperative behavior, less OCB and less willingness to compromise [68]. Even though the cynic reaction may help to lower individual unease and hinder expressive anger reactions [24], it decreases the likelihood of problem solving actions and has therefore been interpreted as basis for resistant behavior [69], or as resistance-strategy [70]. The work of Maslach and Leiter [71], who interpret cynicism as one elementary factor for burnout and as potentially increasing social conflict, should also be mentioned here.

Cynicism can be prevented by social support [72], e.g., the direct support by the manager. Especially important during change are continuous communication processes regarding change progress. Continuous communication aims to build trust [62] especially when focusing on employees with lower status—who show higher humor scores in our study. 

#### 3.3.6. The Role of the Habitual Anger Reaction Submission

**Figure 2:**
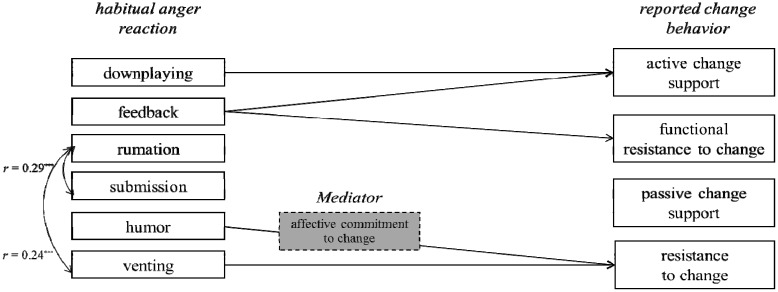
Overview of the results.

We found a significant (*p* < 0.001) correlation of submission with calculative commitment (*r* = 0.205). Further analysis that go beyond the scope of this paper show that the influence on mere compliance is fully mediated by calculative commitment to change (*z* = 3.07, *p* < 0.01). So we claim further research needs to elaborate the ambivalent role of submission in the context of high risk awareness for individuals in organizations. Figure 2 provides an overview over the main results reported in this paper. 

#### 3.3.7. Limitations

Even though current research shows that the habitual anger reaction serves as a valid predictor for actual anger reaction [19], it should be taken into account that the present study is based on cross-sectional data and therefore we could not assess causality. Longitudinal studies are requested to gain an in-depth understanding of the interdependencies aforementioned. This would also put into perspective the retrospective estimation of anger state and context factors of the change event. Another critical aspect of our study is the use of self-report data for behavior during change. More objective data (e.g., manager-ratings) could enhance the quality of our data, but bears the risk of reduced confidentiality which in turn lowers the quality of self-reported behavior during change [8]. A second thought regarding the behavior during change scale: We considered resistance to change to be independent from supportive change behavior which is different from existing conceptualizations of change behavior [37]. This approach takes the likely existence of ambivalence in the context of organizational change into account, but also the multidimensionality of resistance to change. Our new dimension, functional resistance to change, leads in this direction. Nevertheless, the change behavior scale needs further validation and differentiation regarding other aspects of resistance to change (e.g., passive resistance or even compliance).

## 4. Conclusions

Our study encourages a differential approach on determinants of change behavior without neglecting situational variables. One aspect we would like to underline while concluding is the importance of the perceived turbulence for commitment to change and also for anger state. Above all during continuous change, perceived turbulence can reflect past change experiences which are important for the understanding of actual commitment and behavior. We consider this approach to be more promising than the idea of a generalized, stable disposition for resistance to change [73]. This leads us to the last research question: How can we add to a differentiated conceptualization of resistance to change? Based on our results, we need to forbear from conceptualizing resistance to change as antipode to change support or extra-role-performance. On the contrary, resistance to change integrates functional and dysfunctional, active and passive, deviant and adaptive aspects. Our understanding of resistance to change allows the integration of learned resistance dispositions, eventually fostered by specific personality traits, and also situative resistance fostered by characteristics of the change. This differentiated conceptualization of resistance to change needs further research to inspire further implications for applied change management and to add to the theoretical background.
